# Effect of remifentanil infusion rate on stress response in orthopedic surgery using a tourniquet application

**DOI:** 10.1186/1471-2253-13-14

**Published:** 2013-07-10

**Authors:** Taketo Shinoda, Wakako Murakami, Yasuo Takamichi, Hiroki Iizuka, Masaaki Tanaka, Yuto Kuwasako

**Affiliations:** 1 Department of Anesthesiology, Showa University Fujigaoka Hospital, 1-30 Fujigaoka, Aoba-ku, Yokohama, Kanagawa 227-8501, Japan

**Keywords:** Tourniquet pain, Remifentanil, Stress response

## Abstract

**Background:**

Currently, in the field of general anesthesia, balanced anesthesia in combination with analgesic, hypnotic, and muscle relaxant is commonly used. Remifentanil is the standard analgesic used in balanced anesthesia, and has contributed greatly to reduce the physical stress of the patient during surgery. We compared the stress response suppression effect of remifentanil by measuring stress hormones in 2 groups treated with different analgesic doses in orthopedic surgery using a tourniquet.

**Methods:**

Twenty patients were randomly divided into 2 groups (10 patients each) undergoing maintenance of general anesthesia with 0.25 μg/kg/min remifentanil and sevoflurane (Group A) and 1.0 μg/kg/min remifentanil and sevoflurane (Group B). Hemodynamic changes, adrenocorticotropic hormone (ACTH), cortisol, antidiuretic hormone (ADH), adrenaline (Ad), noradrenaline (NAd), dopamine (DOA), insulin, and blood glucose were measured at the initiation of general anesthesia,10 minutes after the initiation of tourniquet application, and immediately before and 10 minutes after the completion of tourniquet application.

**Results:**

ACTH, cortisol, ADH, Ad, and NAd levels in Group B were significantly lower (ACTH and cortisol: P < 0.01, ADH, Ad, and NAd: P < 0.05) than those in Group A. No significant differences were noted in DOA, insulin, or blood glucose levels between the groups.

**Conclusion:**

Anesthesia management with high-dose remifentanil (1.0 μg/kg/min) suppressed intraoperative tourniquet pain-induced stress hormone release, suggesting its usefulness in stabilizing hemodynamics.

**Trial registration:**

JMA-IIA00094

## Background

In orthopedic surgery using a tourniquet application (TA), tourniquet pain increases as the duration of avascularization becomes prolonged, and intraoperative hemodynamic variations increase, such as elevations in blood pressure and tachycardia, which may make anesthesia management difficult. Generally, tourniquet pain-induced stress reactions, such as elevations in blood pressure, undergo symptomatic treatment, such as the administration of additional analgesics and antihypertensive drugs, but anesthesia management with a low risk of adverse events is necessary to reduce the risk of complications. When strong stressful stimulation, such as tourniquet pain, is expected, anesthesia management at a higher analgesic level than a conventional level may facilitate anesthesia with stable hemodynamics, which is gentler on the patient’s body.

Remifentanil can be readily administered at a sufficient analgesic dose during surgery because the analgesic dose is highly adjustable and the drug does not accumulate due to a short context-sensitive half-time [1,2].

In this study, we investigated the stress response suppression effect of remifentanil by measuring and comparing the hemodynamics and stress hormone levels of patients treated at 2 different analgesic doses in orthopedic surgery using a TA.

## Methods

This study was performed after approval by the Ethics Committee of Showa University Fujigaoka Hospital and the patient gave informed consent.

Subjects were 20 patients aged over 16 years old with the American Society of Anesthesiologists (ASA) physical status I-II undergoing orthopedic surgery of the lower limbs (knee joint) using a TA. Patients with the following conditions were excluded: poorly controlled hypertension, abnormal glucose tolerance with 5.5% or higher HbA1c, past medical history of steroid hormone administration, requirement of intraoperative blood transfusion, past medical history of narcotic analgesic administration, and complications of Parkinson’s disease.

As this is an exploratory study, we established the target number of patients, assuming that blood glucose levels may indirectly represent analgesic levels. According to previous data from patients who underwent cardiac surgery, blood glucose levels, which may become a parameter of the analgesic level, were 118 mg/dL in the 0.25 μg/kg/min remifentanil group and 92 mg/dL in the 1.0 μg/kg/min remifentanil group [3]. As the mean blood glucose level is approximately 120 mg/dL, based on the results of a clinical observation of anesthesia control, we assumed that the mean blood glucose levels in Groups A and B were 120 and 90 mg/dL, respectively, in this study. When establishing the standard deviation as 20 mg/dL, a 0.05 level of significance (paired) and 80% power of detection in the two groups, 16 patients (n = 8 per group), were estimated to be necessary for the *t*-test to detect a difference in the mean between the groups. Considering dropout cases, we established the number of patients to be registered as 20 (10 per group)

This study was designed as a randomized controlled trial. Subjects were registered in a patient allocation table by block randomization, and were allocated to either Group A or Group B for investigation in accordance with this allocation table.

Patients stopped eating and drinking from 21:00 on the day before surgery and general anesthesia was initiated between 8:30 and 9:00 a.m. in all patients.

After entering the operation room, a venous line using a 20-G needle and an arterial line for blood sampling using a 22-G needle were established in the left forearm and radial artery, respectively.

**Figure 1:**
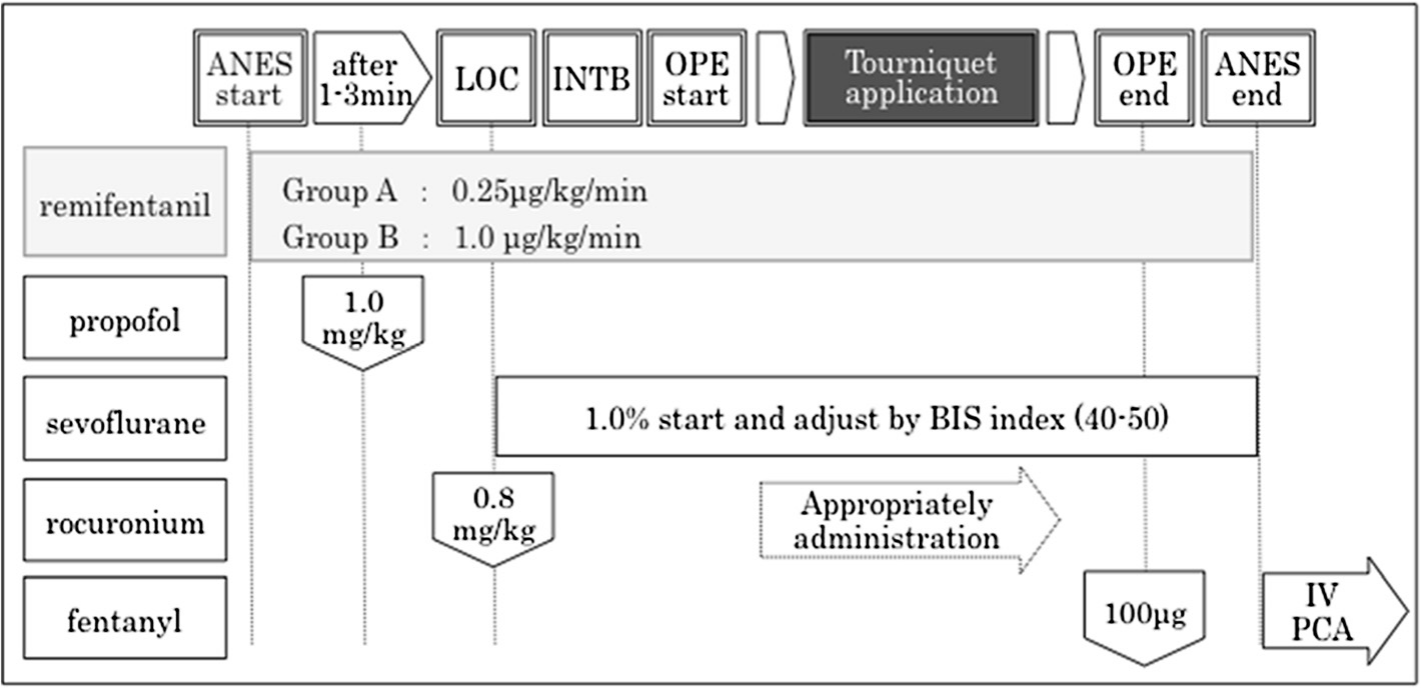
**Anesthetic protocol.** *Case of hemodynamic suppression (bradycardia, hypotension). - systolic blood pressure (SBP) 80 mmHG continued for 1 min: administration of phenylephrine 0.1 mg or ephedrine 8 mg. - Heart rate (HR) 40 beats/min continued for 1 min: administration of atropine 0.5 mg. * Case in stress response (tachycardia, hypertension). - administration of remifentanil 1 μg/kg bolus (0.1 mL per 10 kg body weight). LOC, loss of consciousness. INTB, tracheal intubation. IV-PCA, intravenous patient controlled analgesia.

General anesthesia was induced with 1.0 mg/kg propofol, 0.8 mg/kg rocuronium, and remifentanil (Group A: 0.25 μg/kg/min, Group B: 1.0 μg/kg/min), followed by mask ventilation with 100% oxygen for 3–4 minutes and then tracheal intubation. Anesthesia was maintained by continuous intravenous administration of remifentanil (Group A: 0.25 μg/kg/min, Group B: 1.0 μg/kg/min) and sevoflurane was administered to adjust the BIS (Bispectral index) value to 40–50. For intraoperative infusion, only extracellular fluid containing 1% glucose was administered (7.0-8.0 ml/hr). For postoperative analgesia, both Groups A and B were treated with intravenous bolus administration of 100 μg of fentanyl 30 minutes before completion of surgery and intravenous patient controlled analgesia with fentanyl was initiated after completion of general anesthesia (Figure 1). Blood was collected from the arterial line at the start of anesthesia (before drug consumption), 10 minutes after TA start, just before TA end, and 10 minutes after TA end, 4 times in total, and adrenocorticotropic hormone (ACTH), cortisol, antidiuretic hormone (ADH), adrenaline (Ad), noradrenaline (NAd), dopamine (DOA), insulin, and blood glucose were measured. Sample analysis was performed by Health Sciences Research Institute East Japan Co., Ltd. (Saitama, Japan). The blood pressure (mean arterial pressure, MAP), heart rate, body temperature, urine volume, blood loss, and average consumption of sevoflurane were recorded as conventional monitoring items. The average consumption of sevoflurane was calculated using the following equation: Amount of sevoflurane used (mL) ÷ 3.3 ÷ vaporizer flow rate (L/min) ÷ duration of administration. All data are presented as the mean ± standard deviation. Statistical analysis was performed using the statistical software JMP©8.0.1(SAS Institute Japan). Between group comparisons were performed employing the chi-square test for nominal scales, unpaired *t*-test and dunnett test for continuous scales, and p < 0.05 was regarded as significant.

## Results

**Figure 2:**
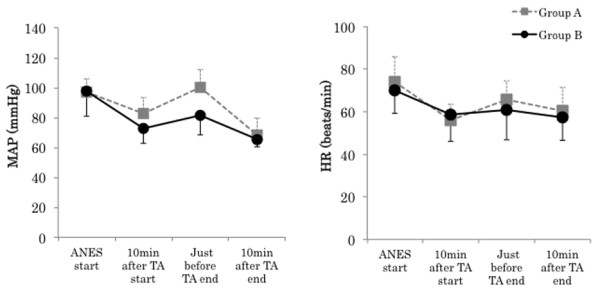
**Changes in mean arterial blood pressure (MAP), and heart rate (HR).** Data are expressed as the mean ± standard deviation. Changes from the baseline (ANES start) values were different (* P < 0.05, ** P < 0.01) between the groups using unpaired *t*-test. Mean value was different (^#^ P < 0.05, ^##^ P < 0.01) than at baseline within the same group using dunnett test. ANES start: Just before drug consumption for induction of anesthesia. TA: Tourniquet application.

**Figure 3:**
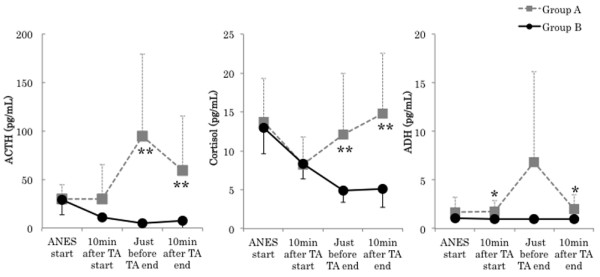
**Changes in adrenocorticotropic hormone (ACTH), cortisol, and antidiuretic hormone (ADH).** Data are expressed as the mean ± standard deviation. Changes from the baseline (ANES start) values were different (* P < 0.05, ** P < 0.01) between the groups using unpaired *t*-test. Mean value was different (^#^ P < 0.05, ^##^ P < 0.01) than at baseline within the same group using dunnett test. ANES start: Just before drug consumption for induction of anesthesia. TA: Tourniquet application. Standard value: ACTH (7.2-63.3 pg/mL), cortisol (4.5-21.1 pg/mL), ADH (0.3-3.5 pg/mL).

**Figure 4:**
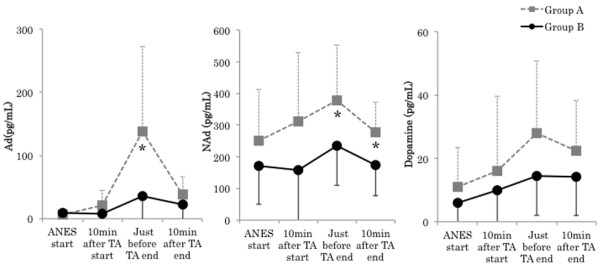
**Changes in adrenaline (Ad), noradrenaline (NAd), and dopamine (DOA).** Data are expressed as the mean ± standard deviation. Changes from the baseline (ANES start) values were different (* P < 0.05, ** P < 0.01) between the groups using unpaired *t*-test. Mean value was different (^#^ P < 0.05, ^##^ P < 0.01) than at baseline within the same group using dunnett test. ANES start: Just before drug consumption for induction of anesthesia. TA: Tourniquet application. Standard value: Ad (<100 pg/mL), NAd (140–450 pg/mL), DOA (< 20 pg/mL).

**Figure 5:**
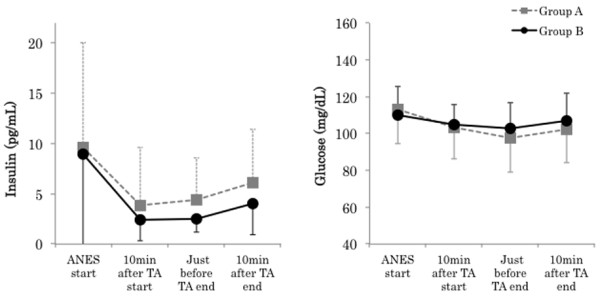
**Changes in insulin and blood glucose.** Data are expressed as the mean ± standard deviation. Changes from the baseline (ANES start) values were different (* P < 0.05, ** P < 0.01) between the groups using unpaired *t*-test. Mean value was different (^#^ P < 0.05, ^##^ P < 0.01) than at baseline within the same group using dunnett test. ANES start: Just before drug consumption for induction of anesthesia. TA: Tourniquet application. Standard value: insulin (2.2-12.4 pg/mL), blood glucose (70–109 mg/dL).

There were no significant differences in the following surgical background items: age, gender, height, body weight, ASA physical status, duration of anesthesia, operation time, duration of TA, infusion volume, and blood loss. No significant difference was noted in intraoperative total infusion volume, but intraoperative urine volume in Group B (740.0 ± 247.6 mL) was significantly larger than that in A (250.0 ± 150.9 mL) (P < 0.01) The average consumption of sevoflurane in Group A (1.3 ± 0.1%) was significantly higher than that in B (1.0 ± 0.1%) (P < 0.01) (Tables 1 and 2). Mean arterial blood pressure (MAP) in Group A was significantly higher than just before TA end (Group A: 100.4 ± 11.9, Group B: 81.6 ± 13.4 mmHg, P < 0.05). The course of the heart rate was similar in the 2 groups, showing no significant differences throughout surgery (Figure 2). There were no significant differences in the BIS value and body temperature between the groups. The highest ACTH level was noted just before TA end in Group A. In contrast, it decreased with time in Group B. A significant difference was noted between the groups just before TA end (Group A: 95.1 ± 84.2, Group B: 4.9 ± 1.6 pg/ml, P < 0.01) and 10 min after TA end (Group A: 59.7 ± 55.7, Group B: 7.7 ±10.2 pg/ml, P < 0.01). Cortisol levels were similar between the 2 groups until 10 min after TA start, but cortisol levels in Group A became higher than those in B and continued to rise thereafter. A significant difference was noted between the groups just before TA end (Group A: 12.1 ± 7.8, Group B: 4.9 ± 1.5 μg/dl, P < 0.05) and 10 min after TA end (Group A: 14.8 ± 7.8, Group B: 5.2 ± 2.4 μg/dl, P < 0.01). ADH levels rose with the avascularization time in Group A, but no rise was noted in Group B. No significant difference was noted between the groups (Figure 3). Ad levels tended to rise in both groups, but the rise in Group B was smaller than that in Group A. A significant difference was noted between the groups 10 min after TA end (Group A: 21.4 ± 23.4, Group B: 7.2 ±8.3 pg/ml, P < 0.05) and just before TA end (Group A: 137.6 ± 135.0, Group B: 35.4 ± 69.9 pg/ml, P < 0.05). The variation pattern of NAd and DOA levels was similar throughout surgery, but both levels in Group B were lower than those in Group A, but this difference was not significant (Figure 4). The variation pattern of insulin levels was similar throughout surgery, but insulin levels in Group B were lower than those in Group A. No significant difference was noted between the groups. No significant difference was noted in blood glucose levels between the groups throughout surgery (Figure 5). No nausea, vomiting, or shivering occurred after surgery in any patient. All patients safely awakened and were extubated in the operation room. Memories during surgery were confirmed the following day, based on which no intraoperative awareness was suspected in any patient of either group.

**Table 1:** Demografic characteristics of the study groups

	**Group A**	**Group B**	**P value**
	**(0.25 μg/kg/min)**	**(1.0 μg/kg/min)**	
Number of patients	10	10	-
Gender (male/female	5/5	5/5	1.0
Age (year)	49.4 ± 27.2	45.6 ± 30.1	0.8
Height (cm)	162.9 ± 10.9	160.9 ± 10.5	0.7
Body weight (kg)	62.1 ± 10.3	60.9 ± 13.7	0.8
ASA Physical status (1/2)	5/5	6/4	0.7

**Table 2:** Comparison of surgery and anesthesia features between the study groups

	**Group A**	**Group B**	**P-value**
	**(0.25 μg/kg/min)**	**(1.0 μg/kg/min)**	
Surgery			0.8
Total knee replacement	5	4	
Arthroscopic anterior cruciate ligament reconstruction	4	4	
Arthroscopic meniscal resection	1	2	
Anesthesia time (min)	172.0 ± 23.6	170.0 ± 21.2	0.8
Operation time (min)	125.0 ± 23.3	123.0 ± 21.0	0.8
Tourniquet application time (min)	96.2 ± 5.7	100.2 ± 13.3	0.4
Fluid volume (mL)	1325.0 ± 178.3	1340.0 ± 269.6	0.9
Urine volume (mL)	250.0 ± 150.9	740.0 ± 247.6	< 0.001**
Blood loss (mL)	50.5 ± 56.0	47.5 ± 52.1	0.9
Average consumption of sevoflurane (%)	1.3 ± 0.1	1.0 ± 0.1	< 0.001**

** Urine volume and average consumption of sevoflurane were significantly higher in Group B than Group A.

## Discussion

The recent objective of anesthesia management is stress-free early recovery after surgery [4]. Surgical stress is closely related to catecholamine synthesis and secretion by the adrenal medulla, and an increase in catecholamine secretion has been reported to inhibit cellular immunity [5]. It has also been reported that inhibition of stress reactions prevented surgical stress-induced perioperative reductions in immune function [6].

Surgical stress elevates the levels of stress hormones (ACTH, cortisol, ADH, Ad, NAd, and DOA) and inflammatory cytokines (TNF-α, IL-1, IL-2, and IL-6) in the body [7]. These promote insulin resistance, gluconeogenesis, and glycolysis and impair insulin secretion, resulting in intraoperative stress-induced hyperglycemia [8]. Intraoperative hyperglycemia has been reported to be an independent risk factor of severe adverse inhospital outcomes (Odds ratio 7.2) [9] and has been suggested to influence mortality [10]. It has also been suggested that ACTH and cortisol release are involved in the development of deep vein thrombosis and pulmonary embolism [11]. Based on these findings, inhibition of stress-induced hyperglycemia and stress hormone release by intraoperative anesthesia management may be useful in improving patient outcomes. It has already been reported that stress hormone and cytokine release and intraoperative hyperglycemia were inhibited by anesthesia management using remifentanil [3,12-14]. The strong analgesic action of high-dose (1.0 μg/kg/min) remifentanil may have suppressed tourniquet pain-induced stress stimulation, stabilized circulatory dynamics, and significantly inhibited stress hormone release in our study.

On the other hand, no significant difference was noted in blood glucose levels between the groups and no stress-induced hyperglycemia occurred, suggesting that insulin secretion was maintained at a higher level in Group A than that in Group B, although the difference was not significant, avoiding hyperglycemia in Group A.

However, in a study in which anesthesia with sevoflurane alone and that with remifentanil and propofol anesthesia were compared in patients during open hysterectomy, stress hormone and blood glucose levels were lower in the TIVA group [9]. In a study in which 5 doses (0.25, 1.0, 2.5, and 5.0 μg/kg/min) of remifentanil were compared in pediatric patients during cardiac surgery, cortisol and glucose level elevations were suppressed in the group treated with remifentanil at 1.0 μg/kg/min or higher [3]. In our previous study in which changes in blood glucose levels during laparoscopic surgery were investigated in groups treated with remifentanil at 0.25 and 1.0 μg/kg/min [15], blood glucose levels were significantly rose in the 0.25 μg/kg/min remifentanil treatment group.

These preceding studies suggest that the blood glucose level may serve as an index of surgical stress inhibition. However, the blood glucose level is influenced by various factors, and no correlation was noted between the stress hormone and blood glucose levels, suggesting that tourniquet pain and organ-injuring stress simulation are different.

The increase in urine volume observed in Group B (1.0 μg/kg/min) may have been due to the suppression of ADH secretion because ADH secretion levels were high in Group A (0.25 μg/kg/min) and there was no difference in infusion volumes between the 2 groups.

## Conclusion

Anesthesia management with high-dose remifentanil (1.0 μg/kg/min) suppressed tourniquet pain-induced stress hormone release during orthopedic surgery of the limbs using a TA, suggesting its usefulness in stabilizing hemodynamics. This protocol should also be evaluated in patients treated with various surgeries. It may be difficult to accurately evaluate intraoperative stress unless stress hormones can be measured simply in an operation room at a low cost. As an alternative, the blood glucose level and urine volume may serve as indices of the analgesic effect because these can be measured simply during surgery.

## Abbreviations

ACTH: Adrenocorticotropic hormone; Ad: Adrenaline; ADH: Antidiuretic hormone; ASA: American Society of Anesthesiologists; BIS: Bispectral index; DBP: Diastolic blood pressure; DOA: Dopamine; NAd: Noradrenaline; SBP: Systolic blood pressure; TA: Tourniquet application.

## Competing interests

The authors declare that they have no competing interest.

## Authors’ contributions

TS: Designing the study, giving informed consent to patients, collecting and analyzing data, writing the manuscript. WM: Collecting data. YT: Collecting data. HI: Collecting data. MT: Analyzing data. YK: Intellectual contribution and supervision. All authors read and approved the final manuscript.

## Pre-publication history

The pre-publication history for this paper can be accessed here:

http://www.biomedcentral.com/1471-2253/13/14/prepub
